# Whether or wither some specialties: a survey of Canadian medical student career interest

**DOI:** 10.1186/1472-6920-9-57

**Published:** 2009-09-04

**Authors:** Ian M Scott, Bruce J Wright, Fraser R Brenneis, Margot C Gowans

**Affiliations:** 1 Department of Family Practice, University of British Columbia, 300-5950 niversity Boulevard, Vancouver, BC V6T 1Z3, Canada; 2 Department of Family Medicine, University of Calgary, 3330 Hospital Dr. NW, Calgary, AB T2N 4N1, Canada; 3 Department of Family Medicine, University of Alberta, 2-65 Zeidler Ledcor Building, University of Alberta Edmonton, Alberta, T6G 2X8, Canada; 4 Department of Family Practice, University of British Columbia, 300-5950 University Boulevard, Vancouver, BC V6T 1Z3, Canada

## Abstract

**Background:**

Given the looming shortage of physicians in Canada, we wished to determine how closely the career preference of students entering Canadian medical schools was aligned with the current physician mix in Canada.

**Methods:**

Career choice information was collected from a survey of 2,896 Canadian medical students upon their entry to medical school. The distribution of career choices of survey respondents was compared to the current physician speciality mix in Canada.

**Results:**

We show that there is a clear mismatch between student career choice at medical school entry and the current specialty mix of physicians in Canada. This mismatch is greatest in Urban Family Medicine with far fewer students interested in this career at medical school entry compared to the current proportion of practicing physicians. There are also fewer students interested in Psychiatry than the current proportion of practicing physicians.

**Conclusion:**

This mismatch between the student interest and the current proportion of practicing physicians in the various specialities in Canada is particularly disturbing in the face of the current sub-optimal distribution of physicians. If nothing is done to correct this mismatch of student interest in certain specialities, shortages and misdistributions of physicians will be further amplified. Studies such as this can give a window into the future health human resources challenges for a nation.

## Background

Even though Canada has more physicians per capita than many nations, reductions in the ratio of physicians to patients combined with an aging population is expected to have implications for the health care system.[1] Between 1993 and 2000 alone, the physician to population ratio dropped by 5.1%.[1] Because of the changing demographics and practice patterns of physician providers, even if we were to maintain the same number of physicians, there would be fewer doctor-hours to provide care to patients. [2-6]

In Canada, the number and specialty of physicians is determined by numerous factors including government policies, undergraduate and postgraduate training opportunities, immigration and emigration of providers, gender and age distribution of providers, remuneration incentives and disincentives and the work life cycle of physicians among a host of factors. [7-14] A significant component of the Canadian medical education system is the Canadian Residency Matching Service--CaRMS. CaRMS is a not-for-profit organization that works with the medical education community, medical schools and students, to provide an electronic application service and a computer match for entry into postgraduate medical training that serves all medical students and all postgraduate programs throughout Canada.[15] Canadian medical school training is 4 years at all schools in Canada except two schools where the training lasts 3 years. Students then enter the CaRMS match at the end of this undergraduate medical training. Residencies in Canada for Family Medicine are typically 2 years and for the other specialties they range from 4-5 years. All Canadian medical graduates wishing to enter a residency in Canada participate in CaRMS. The residency match is viewed by medical students as a high stakes activity and students devote significant attention to ensuring that they receive their top choice in the CaRMS match.[16] In 2008, 91% of Canadian medical graduates were matched to their top discipline at the end of the first round of the match.[17]

At present, there is an over-supply of postgraduate medical training positions in Canada for Canadian medical graduates. Even with the inclusion of international medical graduates (IMGs) into the CaRMS process, there were 121 unfilled residency spots in Canada at the end of the second CaRMS match in 2008.[18] As a result, the career preference of Canadian medical graduates has an important impact on the mix of practicing physicians.[19] There is currently no clear national strategy in Canada for discussing the mix of residency positions that should be made available to medical students. This lack of a national strategy at the post-graduate training level coupled with an excess of residency positions means that medical students have a significant role in determining the future mix of providers. Canada is not unique in having a matching service nor an oversupply of postgraduate positions for students which places student choice as an important factor in the mix of future providers. [20,21] Other regions are also moving towards a coordinated national match.[22]

While students do not apply for residency positions until their final year of medical school, for many, the choice between primary care and specialty medicine has been made prior to commencement of or early in medical school. [23-26] For some students, they already have a specific specialty firmly in mind prior to medical school entry.[27] While career choices at medical school entry are clearly mutable, it is clear that these early choices are predictive of ultimate career direction.

While the current mix of physician providers may not be optimal for our health care system needs, it nonetheless represents the structure and function of our current system. To further understand what influence student choice will have on the future of the Canadian Health Care System, we wished to determine how closely the career preference of students entering medical school was aligned with the current physician mix in Canada.

## Methods

### Setting

As part of multi-year, multi-centre investigation [28-30] into medical student career choice, we have been surveying student career aspirations at eight Canadian medical schools. Students were surveyed at the University of British Columbia, University of Calgary, University of Alberta, University of Ottawa, University of Toronto, University of Western Ontario, Queen's University and McMaster University. These Universities volunteered from among 13 English speaking medical schools operating at the beginning of this study.

### Subjects

International students were excluded from the study. From the remaining students, a cohort of 3,225 students entering the above schools between 2002 and 2005 (depending on the school over a one to four year period), were asked to complete a survey on career choice.

### Procedure and Instrument

During the first few weeks of medical school, students were asked to identify and rank their top three career choices. Students were offered the following list of possible career choices: Emergency, Urban Family Medicine, Rural Family Medicine, Internal Medicine, Obstetrics and Gynaecology, Paediatrics, Psychiatry, Surgery, and Other (a write in option). These career choices were pre-selected as it was felt they would be clear and distinct for most students in their first few weeks of medical studies. Urban and Rural Family Medicine was chosen to further understand the issue of the future of primary health care in rural and remote communities. A series of other attitudinal and demographic questions were asked that are not included in this study. Two reminders were sent by email to students who did not respond. For the purposes of this study, only the students' top career choice was considered.

Data analysis was performed using SPSS version.14.0 (SPSS Inc. Chicago, USA). Descriptive analysis was used to present student career interest on entry to medical school. Comparisons between the proportion of students indicating each major career choice and the proportion of Canadian physicians currently practicing in that specialty [31] were made using the chi-square test statistic. In all cases, a result was considered significant for p < 0.05.

Ethical approval was received by the appropriate university ethics boards at all participating schools.

## Results

The overall response rate on this entry survey was 89.8% (2,896/3,225). Eighteen students (0.6%) were excluded from analyses due to our inability to classify their write in career choice (i.e. sports medicine, interpretive medicine, performance art medicine). In addition, 139 students (4.8%) failed to specify their top career choice or stated that they did not have a top career choice and were thus excluded from analysis. A final sample of 2,739 students with valid responses was analyzed.

Valid survey respondents ranged in age from 19 to 49 years, with a median of 23.0 years and a mean of 24.0 years. A majority of responents were female (56.7%) and single (71.1%) and had entered medical school from a science background (91.7%). Most (75.7%) came from families where their most educated parent had a university education, 41.2% having close family or friends practicing medicine and 20.4% having spent a majority of their childhood living in a rural community (self defined).

The most popular career choice among students at medical school entry was internal medicine and associated medical subspecialties (28.9%), closely followed by family medicine (rural and urban combined, 25.9%). The least popular career choice amongst those listed was psychiatry (2.9%) (See Table 1).

**Table 1:** Interest of Medical Students in Specific Careers at MD school Entry

**Specialty**	**n**	**%**	**95% CI**
Internal Medicine and Medical subspecialties	791	28.9	27.2 - 30.6

Surgery and Surgical subspecialties	478	17.5	16.1 - 18.9

Pediatrics	418	15.3	14.0 - 16.6

Urban Family Medicine	420	15.3	14.0 - 16.0

Rural Family Medicine	291	10.6	9.4 - 11.8

Emergency	158	5.8	4.9 - 6.7

Obstetrics and Gynecology	103	3.8	3.1 - 4.5

Psychiatry	80	2.9	2.3 - 3.5

TOTAL	2739	100.0	

The career interests expressed by students differed significantly from the current mix of full time equivalent physicians for all specialties (p < 0.005) (Table 2).

**Table 2:** Current Physician Mix in 2003-2004 compared to MD Student Interest

**Discipline**	**Current Physicians**		**Entering MD students**	
	
	**n**	**%**	**%**	
Urban Family Medicine	26324	43.4	15.3	*X*^2 ^= 878.30, df = 1 p < 0.001

Internal Medicine and medical subspecialties	14969	24.7	28.9	*X*^2 ^= 25.72, df = 1 p < 0.001

Surgery and surgical subspecialties	6131	10.1	17.5	*X*^2 ^= 163.03, df = 1 p < 0.001

Rural Family Medicine	4962	8.2	10.6	*X*^2 ^= 21.39, df = 1 p < 0.001

Psychiatry	4,014	6.6	2.9	*X*^2 ^= 60.15, df = 1 p < 0.001

Pediatrics	2,152	3.6	15.3	*X*^2 ^= 1073.22, df = 1 p < 0.001

Obstetrics and Gynecology	1,593	2.6	3.8	*X*^2 ^= 14.57, df = 1 p < 0.001

Emergency Medicine	467	0.8	5.8	*X*^2 ^= 852.01, df = 1 p < 0.001

Total	60612	100	100	

## Discussion

According to the Canadian Institute for Health Information (CIHI), the number of active physicians in Canada is 60,612. [31] It is apparent in comparing student career choice at medical school entry with the current mix of active physicians that there is mismatch between student interest and the current physician workforce (See table 2). While career interest at entry to medical school does not definitively indicate career choice on graduation, there is increasing evidence of a strong association. Studies have demonstrated that from 45% to 70% of students predict their ultimate specialty choice at medical school entry. [23-26]

This mismatch is greatest for Urban Family Medicine for which the proportion of students interested in this career choice at medical school entry is 28.1% points below the current proportion of physicians practicing in this discipline. There is also discordance between student career choice and the current workforce mix in Psychiatry with student interest 3.7% points below the current proportion of physicians practicing in this discipline. There is a greater interest among entering medical students in the careers of Emergency Medicine (5.0% points greater interest), Internal Medicine and medical subspecialties (4.2% points greater interest), Surgery (7.4% points greater interest) and Paediatrics (11.7% points greater interest) compared to the current proportion of physicians practicing in these areas. There is a near match of the student interest in Obstetrics and Gynaecology (1.2% points greater interest) and Rural Family Medicine (2.4% points greater interest) compared to the current proportion of physicians practicing in these disciplines.

It is likely that the 5% surplus interest in emergency medicine is not an accurate representation of excess interest compared to the current workforce of emergency providers in Canada. CIHI classifies all certificants of the College of Family Physicians of Canada as Family Physicians {both CCFP and CCFP (EM)}. As of May 9 2008, there were 1,796 physicians with a CCFP (EM) designation in the College of Family Physicians of Canada Membership Database. [Personal Communication from Sarah Scott sscott@cfpc.ca, National Physician Survey & Janus Project Coordinator, College of Family Physicians of Canada to Ian Scott, May 9, 2008)] Chan [32] estimates that 50% of physicians with CCFP (EM) designation practice primarily emergency medicine. Others have found even higher numbers CCFP (EM) physicians practicing primarily emergency medicine with less than 20% engaged in a blended family/emergency medicine practice.[33] In addition, in many rural or remote jurisdictions physicians practice emergency medicine without any form of certification. Thus, the actual excess interest in emergency medicine compared to actual providers of emergency care may be smaller than is observed if one were able to compare student interest with the actual numbers of physicians providing emergency care across Canada.

The impact of the number of international medical graduates (IMGs) who enter the system at the level of the CaRMS match is modest but growing. During the 2008 CaRMS match, IMGs (who were Canadian Citizens or Landed Immigrants) accounted for 14.2% (353) of the total residency positions filled (2478). [Personal Communication email from Jim Boone jboone@carms.ca General Manager and COO, CaRMS to Ian Scott January 22, 2009] Overall the national percentage of IMGs within the Canadian medical workforce has declined slightly from 23.1% in 2000 to 22.3% in 2004.[34]

Our study shows nearly the same proportion of graduates interested in practicing rural family medicine as are currently providing service in rural communities. However, this is already an under-serviced geographical area. Rourke estimates that an additional 1175 family physicians are required to bring the family physician-population ratio to the same level as the Canadian average.[35]

This study begs the question: "What is the appropriate mix of physician specialities for Canada?". While a number of organizations, commissions and reports [36-41] have looked at this issue, there are as yet no clear recommendations. There have been calls for greater systematic centralized physician data collection but these calls have not yet been heeded.[42] Recently the Canadian Medical Association Journal requested human resources data from the College of Family Physicians of Canada and all 47 specialty groups registered with the Royal College of Physicians and Surgeons of Canada.[43] Only 27 groups responded to the request and of those who responded, only 13 had done studies over the last decade on the health human resources issues in their discipline.[43] Of those that had done studies, only six could quantify existing shortages of physicians in their specific discipline. With over 4,000 physicians planning to retire or leave practice in the next 2 years, the current number of Canadian medical graduates barely fills this void particularly given the entering cohort's career intentions.[8]

Given that there is evidence that student interest at career entry is associated with a student's ultimate career choice [23-27], this data gives insight into not only the future career aspirations of medical students but the possible future structure and function of the Canadian Health Care System. This study therefore heralds a future Canadian Health Care system populated by increasing numbers of specialists, decreasing numbers of urban family physicians and the on-going insufficient number of rural family physicians.

Since medical educators have a social responsibility to be accountable for the health care needs of the population they serve [44], it may be time to selectively recruit students and to modify medical school curricula in such a way as to better meet the future needs of Canada's population. An alternative solution would be to limit the number of postgraduate positions in certain disciplines to drive students towards the required career choices. Such a strategy may result in many students finding themselves in careers they are not suited for or students choosing to move to other jurisdictions for postgraduate training to achieve their desired career choices. Both of these outcomes could have negative implications for the Canadian Health Care System.

This study is limited by not including all medical schools in Canada and while it has surveyed nearly 3000 students from eight Canadian medical schools over a time period of up to 4 years, the results might not be generalizable to the entire country. We will be following these students forward to determine what their ultimate career choice and career matches is. In addition we will seek to understand what factors at medical school entry predict a student's ultimate career choice beyond their stated career interest at the beginning of medical school.

## Conclusion

In light of expected shortages of physicians in Canada and other countries, the career desires of entering medical students can provide insight into the future structure of the health care system. Health planners should heed these student desires and take proactive action in planning the health care system, otherwise countries may get a health care system that medical students have defined for them.

## Competing interests

The authors declare no financial or non-financial competing interests other than they are members of departments of family physicians and wish to ensure a sustainable supply of family physicians for the Canadian health care system.

## Authors' contributions

IS, BW and FB contributed to the study conception and design. MG developed the approach to the statistical analyses of the data. IS wrote the first draft of the manuscript with all authors contributing to the revisions. All authors read and approved the final manuscript.

## Authors' Information

Ian Scott is the Undergraduate Director of Family Practice at the University of British Columbia. Bruce Wright is the Associate Dean Undergraduate Medical Education at the University of Calgary. Fraser Brenneis is the Senior Associate Dean, Education for the Faculty of Medicine & Dentistry at the University of Alberta. Margot Gowans is a social sciences researcher in the Department of Family Practice at the University of British Columbia.

## Pre-publication history

The pre-publication history for this paper can be accessed here:


